# Generation of a toxin/antitoxin-based counterselection marker for *Chlamydia trachomatis*

**DOI:** 10.1128/iai.00537-25

**Published:** 2025-11-18

**Authors:** Eleanor Steiner, Samantha D’Spain, Rachel Ende, Isabelle Derré

**Affiliations:** 1 Department of Microbiology, Immunology, and Cancer Biology, University of Virginia 2358, Charlottesville, Virginia, USA; University of California Davis, Davis, California, USA

**Keywords:** *Chlamydia trachomatis*, counterselection marker, toxin-antitoxin systems, CcdA/CcdB, VapB/VapC, MvpA/MvpT, genetic tools

## Abstract

*Chlamydia trachomatis* is the leading cause of bacterial sexually transmitted infection in the United States. The high rate of asymptomatic cases and absence of a vaccine often leave infections untreated, increasing the risk of serious complications in women, like pelvic inflammatory disease, ectopic pregnancy, and infertility. The generation of *C. trachomatis* mutants is crucial for studying *C. trachomatis* gene function, identifying potential vaccine candidates, and understanding host-pathogen interactions. However, the obligate intracellular nature of the bacteria hinders the development of genetic tools for mutagenesis. Counterselectable markers are effective systems for selecting bacterial mutants; however, such systems have yet to be optimized for use in *C. trachomatis*. In this study, we created a toxin-antitoxin (TA) system-based counterselection marker. Two TA systems were tested, toxin CcdB and its antitoxin CcdA (CcdAB), and toxin MvpT and its antitoxin MvpA (MvpAT). For each system, the antitoxin was expressed from a constitutive promoter, while the toxin was controlled by an inducible promoter. We first showed that, in *Escherichia coli*, toxin induction in both TA systems overcame the protective effect of the antitoxin, resulting in growth inhibition. The two systems were subsequently tested in *C. trachomatis*. While the CcdAB system did not significantly inhibit the growth of *C. trachomatis*, the MvpAT system did. Altogether, we have developed an MvpAT-based counterselection system for use in *C. trachomatis*. Implementation of this system will enable more efficient genetic manipulation, facilitating the identification of bacterial virulence factors and advancing translational research toward improved treatment and prevention.

## INTRODUCTION

*Chlamydia trachomatis* is the causative agent of the most common sexually transmitted infection of bacterial origin in the United States. Due to the high rate of asymptomatic infection and the lack of an effective vaccine for prevention, untreated infections are common and associated with serious health concerns in women, including pelvic inflammatory disease, ectopic pregnancy, and infertility (1, 2).

The generation of *C. trachomatis* mutants is critical to the study of *C. trachomatis* gene product function, identification of potential vaccine candidates, and understanding of host-pathogen interactions at the molecular level. However, the obligate intracellular nature of the bacteria has made it difficult to develop genetic tools for mutagenesis and link genotype to phenotype (3, 4). In addition, *C. trachomatis* is an obligate intracellular pathogen that undergoes a unique biphasic developmental cycle, alternating between an infectious and replicative form, within the lumen of a membrane-bound compartment called the inclusion (5). This intracellular lifestyle further complicates genetic manipulation. While the genetic toolkit for mutagenesis of *C. trachomatis* has expanded in the last decade and a half (6–13), methods like allelic exchange mutagenesis remain challenging, as clones that have undergone allelic exchange only represent a small portion of total transformants.

In many bacteria, isolating clones that have undergone allelic exchange via two episodes of homologous recombination is facilitated by pairing selection and counterselection markers (14). The *Bacillus subtilis sacB* gene is a commonly used counterselection marker that has been successfully employed in several bacteria, including *Legionella pneumophila* (15), *Pseudomonas aeruginosa* (16), *Mycobacterium* (17), and *Klebsiella* (18). The *sacB* gene encodes a levansucrase that converts sucrose to levans, a toxic substance to bacteria (19, 20). Thus, bacterial cell death and counterselection are induced when gram-negative bacteria encoding the *sacB* gene are propagated in the presence of sucrose (20). However, the obligate intracellular lifestyle of *C. trachomatis* and the several membrane barriers that sucrose would have to cross (host plasma membrane, inclusion membrane, and bacterial outer membrane) to reach the bacterial periplasm may be a significant challenge, preventing the use of the *sacB* counterselection system in this obligate intracellular pathogen. Therefore, bypassing the physical barriers of the plasma and inclusion membranes is an important consideration in the design of an effective counterselection system for *C. trachomatis*.

Naturally occurring toxin-antitoxin (TA) systems are widespread among prokaryotes (21, 22). In these systems, the toxin targets essential processes, such as DNA replication, transcription, or translation (21, 22). The antitoxin is encoded with the toxin and neutralizes its activity under steady-state conditions. In response to specific stimuli, such as plasmid loss, environmental stress, or phage infection, the antitoxin is degraded or inactivated, allowing the toxin to act. This activation can promote persistence, inhibit phage propagation, or eliminate damaged bacteria, thereby providing a competitive advantage at the population level (21, 22).

**Fig 1:**
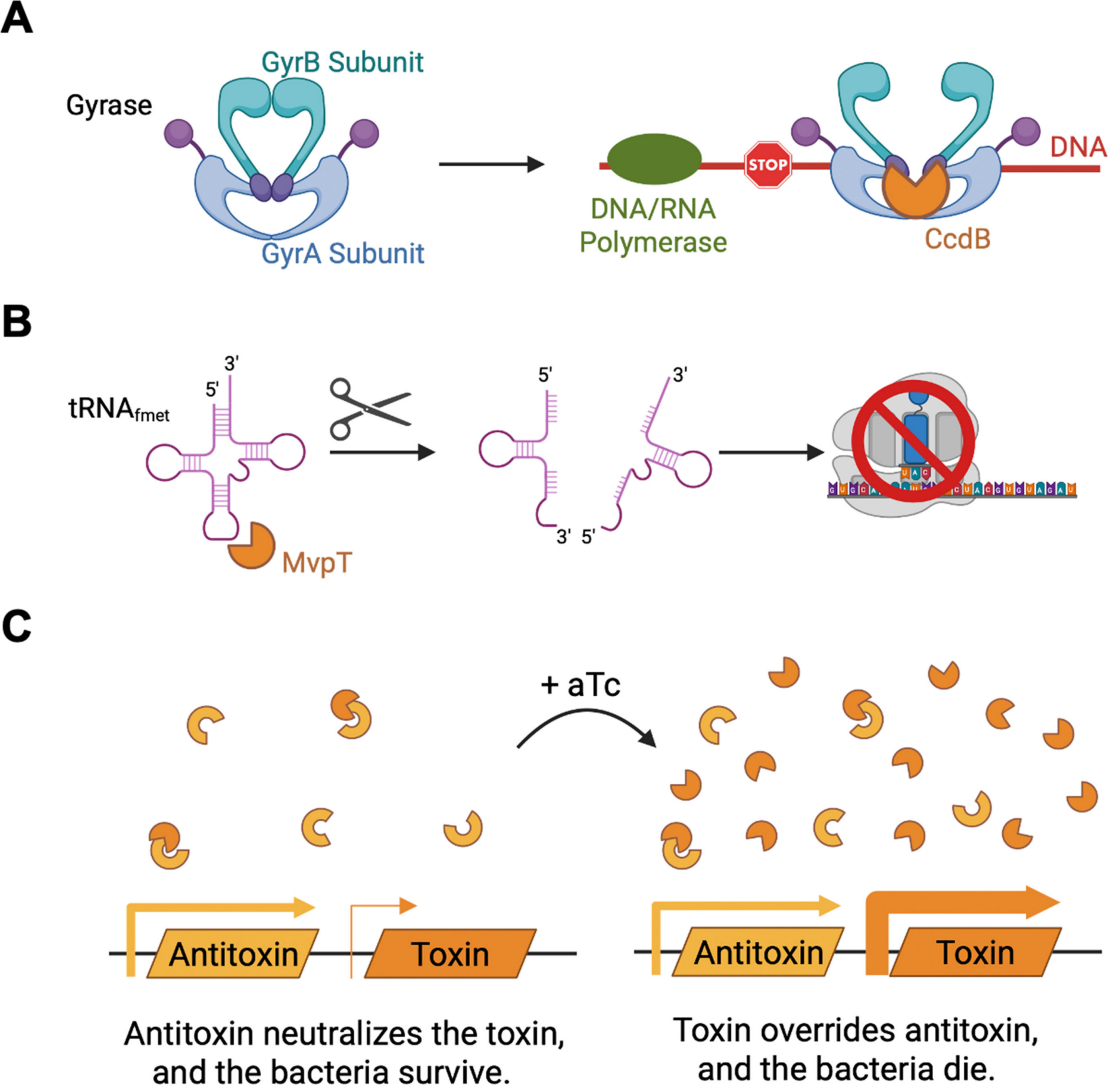
Graphical representation of the mode of action of the CcdB and MvpT toxins, and of the engineered TA module. (**A**) Mechanism of action of the CcdB toxin. During DNA and RNA polymerization, the DNA gyrase, which is made of two subunits, GyrA (blue) and GyrB (teal), generates double-strand DNA breaks. The CcdB toxin (orange packman) binds to the GyrA subunit, locking the gyrase in place, thus preventing DNA transcription and replication. Model adapted from reference 28. (**B**) Mechanism of action of the MvpT toxin. MvpT (orange packman) cleaves tRNA_fmet_ (purple) between nucleotides +38 and +39, thereby preventing the initiation of translation. Model adapted from reference 29. (**C**) The antitoxin is cloned under a constitutively active promoter (yellow rectangles and arrows). The toxin is cloned under an anhydrotetracycline (aTc) inducible promoter (orange rectangles and arrows). The thickness of the arrows indicates the strength of the promoter. At steady state (left), little to no toxin (orange packman) is produced, and its effect is counteracted by an excess of antitoxin (yellow incomplete circle). In the presence of aTc (right), toxin (orange packman) expression is induced, while antitoxin expression remains unchanged (yellow incomplete circle), resulting in a change in the toxin to antitoxin ratio and an increase of free toxin. Cartoons were generated using BioRender.

One such TA system is the toxin CcdB and its antitoxin CcdA (CcdAB) TA system, which is composed of the toxin CcdB and its corresponding antitoxin, CcdA. The CcdB toxin binds the GyrA subunit of the topoisomerase II DNA gyrase, trapping the gyrase in complex with the DNA and inducing double-stranded DNA breaks (23) (Fig. 1A). This action is counteracted by the antitoxin CcdA, which binds to CcdB and releases it from the gyrase (24). Similarly, the VapBC TA system, also known as toxin MvpT and its antitoxin MvpA (MvpAT), is composed of the toxin VapB/MvpT and its respective antitoxin VapC/MvpA. The toxin VapB/MvpT is a PIN domain ribonuclease (25) that cleaves the initiator tRNA^fmet^ (Fig. 1B). Cleavage occurs at positions +38 and +39 (26), which is conserved in the fMet tRNA sequence of *Escherichia coli*, *Shigella flexneri*, *and C. trachomatis* and located at the boundary of the stem loop anticodon of the tRNA (Fig. S1), thus inhibiting translation and preventing bacterial growth (26). When present, the antitoxin VapC/MvpA binds to and neutralizes the VapB/MvpT toxin (27).

Among others, the CcdAB and VapBC/MvpAT TA systems have been successfully harnessed and manipulated to serve as counterselection systems for genetic manipulation of bacteria. The gyrase poison CcdB enabled counterselection of plasmid integration and generation of markerless allelic replacement in *Vibrio splendidus* and *V. cholerae* (30), and *E. coli* (31). In *E. coli*, CcdB was also applied to chromosomal gene knock-in (32). The tRNAase VapC and its anti-toxin VapB were used for markerless gene disruption in *Pyrococcus yayanosii* (33).

In this study, we hypothesized, engineered, and demonstrated that an endogenously expressed TA system could serve as an efficient and self-contained counterselection marker in *C. trachomatis*, eliminating the need for exogenous factors. Harnessing this TA system will allow for more efficient genetic manipulation of the bacterium, facilitating the identification of bacterial factors important for its virulence and supporting translational research to combat *C. trachomatis* infections.

## RESULTS AND DISCUSSION

### The engineering of inducible CcdAB and MvpAT TA systems

**Fig 2:**
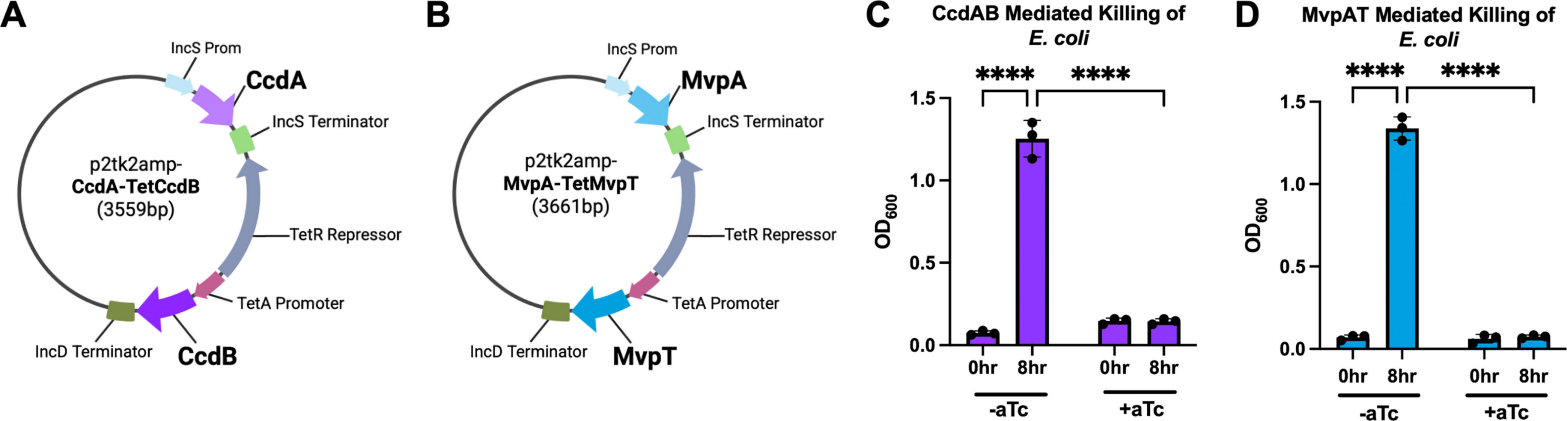
Expression of toxins CcdB or MvpT prevents the growth of DH5α *E. coli*. (**A**) Vector map for p2tk2_Amp_-CcdA-TetCcdB. Starting at the top of the plasmid and moving clockwise: the *incS* promoter (light blue), the antitoxin CcdA (light purple), the *incS* terminator (light green), the TetR repressor (gray), the *tetA* promoter (burgundy), the CcdB toxin (purple), and the *incD* terminator (dark green). (**B**) Vector map for p2tk2_Amp_-MvpA-TetMvpT. Starting at the top of the plasmid and moving clockwise: the *incS* promoter (light blue), the antitoxin MvpA (lighter blue), the *incS* terminator (light green), the TetR repressor (gray), the *tetA* promoter (burgundy), the MvpT toxin (blue), and the *incD* terminator (dark green). (**C and D**) Growth of DH5α *E. coli* harboring the CcdAB (**C**, purple) or MvpAT (**D**, blue) modules over 8 h in the presence (+aTc) or absence (−aTc) of aTc. Data are three independent experiments (*n* = 3) plotted independently and with mean ± SEM, two-way ANOVA with multiple comparison. ****p < 0.0001.

The TA systems were engineered as inducible toxin systems in which, under baseline conditions, the antitoxin is constitutively expressed, while the toxin remains unexpressed and harmless. Upon induction, toxin expression overrides the constitutive expression of the antitoxin, resulting in inhibition of bacterial growth (Fig. 1C). We selected two systems from *S. flexneri*: CcdAB and MvpAT, with MvpAT being a homolog of the VapBC family. For both systems, the antitoxin CcdA or MvpA was cloned under the constitutively active *C. trachomatis incS* promoter (Fig. 2A and B). The toxin CcdB or MvpT was cloned under the anhydrotetracycline (aTc) inducible promoter (Fig. 2A and B). These engineered TA modules were subsequently cloned into the p2tk2_Amp_ plasmid, a minimal plasmid for replication in *E. coli*. To evaluate the effectiveness of the designed system, the two resulting plasmids, p2tk2_Amp_-CcdA-TetCcdB (Fig. 2A) and p2tk2_Amp_-MvpA-TetMvpT (Fig. 2B), were transformed into DH5α *E. coli*.

### The engineered MvpAT and CcdAB TA systems inhibit *E. coli* growth under toxin induction conditions

To evaluate the ability of the engineered CcdAB and MvpAT TA systems to inhibit *E. coli* growth upon induction of toxin expression, spectrophotometry was used to measure the turbidity of *E. coli* liquid cultures in the presence or absence of aTc. Overnight cultures of DH5a *E. coli* containing p2tk2_Amp_-CcdA-TetCcdB or p2tk2_Amp_-MvpA-TetMvpT were diluted in the absence or presence of aTc to achieve an OD_600_ value of ~0.05. After 8 h of incubation at 37°C, the turbidity of the liquid cultures was measured.

In the absence of aTc, *E. coli* encoding the CcdAB TA system proliferated to an average OD_600_ of 1.253 after 8 h of growth (Fig. 2C; purple bars, −aTc, 8 h), effectively reaching stationary phase growth. In comparison, in the presence of aTc, there was no change in the OD_600_ between 0 and 8 h of culture (Fig. 2C; +aTc, 0–8 h). Similar results were observed for the MvpAT TA system, with *E. coli* only reaching stationary phase in the absence of aTc (Fig. 2D; blue bars, −aTc, 8 h).

These results indicate that the CcdB and MvpT toxins were inactive under basal conditions. However, in the presence of aTc, the expression of the respective toxin was induced, leading to significant inhibition of *E. coli* growth, thereby validating the functionality of our designed TA systems.

### The generation of *C. trachomatis* strains encoding the MvpAT and CcdAB TA system

**Fig 3:**
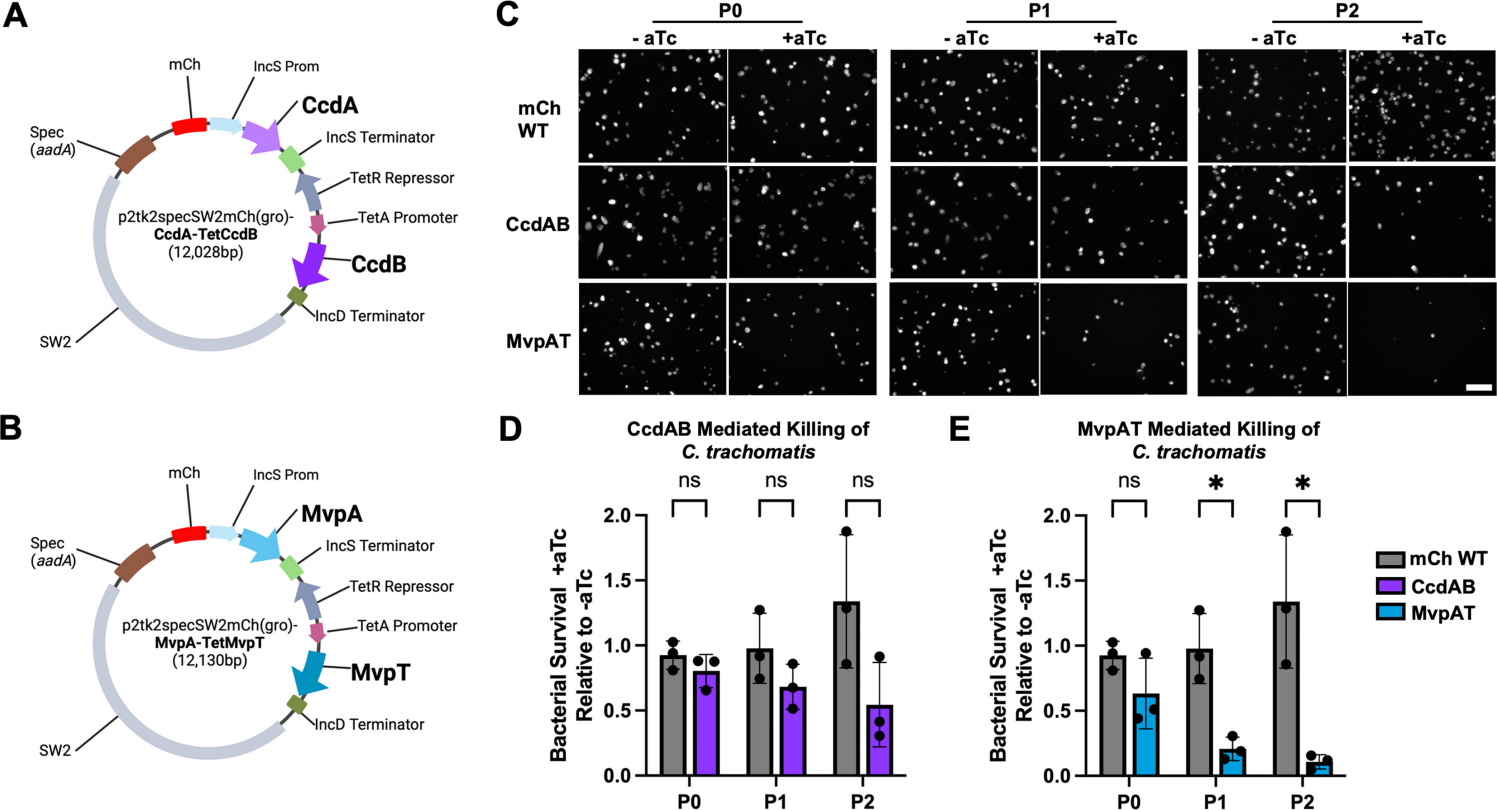
The MvpAT TA system conditionally inhibits *C. trachomatis* propagation. (**A**) Vector map for p2tk2_Spec_SW2mCh(gro)-CcdA-TetCcdB. Starting at the top of the plasmid and moving clockwise: the *incS* promoter (light blue); the antitoxin CcdA (light purple); the *incS* terminator (light green); the TetR repressor (gray); the *tetA* promoter (burgundy); the CcdB toxin (purple), the *incD* terminator (dark green); SW2, which contains the *C. trachomatis* origin of replication (light gray); the *aadA* gene, which confers spectinomycin resistance to the bacteria (brown); and mCherry (red). (**B**) Vector map for p2tk2_Spec_SW2mCh(gro)-MvpA-TetMvpT. Starting at the top of the plasmid and moving clockwise: the *incS* promoter (light blue); the antitoxin MvpA (lighter blue); the *incS* terminator (light green); the TetR repressor (gray); the *tetA* promoter (burgundy); the MvpT toxin (blue); the *incD* terminator (dark green); SW2, which contains the *C. trachomatis* origin of replication (light gray); the *aadA* gene, which confers spectinomycin resistance to the bacteria (brown); and mCherry (red). (**C**) Fluorescence micrographs of HeLa cells infected with either CTL2 mCh WT (top panels), CTL2 p2tk2_Spec_-CcdAB (middle panels), or CTL2 p2tk2_Spec_-MvpAT (bottom panels) in the presence (+aTc) or absence (−aTc) of aTc, 48 h post-infection (P0) and 48 h after one (P1) and two (P2) passages. Each white dot on the fluorescent micrograph is one mCherry-positive *C. trachomatis* inclusion. (**D and E**) Quantification of the number of inclusions as shown in panel **C** normalized to their respective −aTc control. Gray: mCh WT (**D and E**), purple: CcdAB (**D**). blue: MvpAT (**E**). Data are three independent experiments (*n* = 3) plotted independently with mean ± SEM, unpaired parametric *t*-test with Holm-Šídák correction for multiple comparisons; *P < 0.01.

To allow for expression in *C. trachomatis*, the MvpAT and CcdAB TA modules were cloned into the *E. coli-C. trachomatis* shuttle vector p2TK2_Spec_SW2mCh(gro) (34). This vector confers spectinomycin resistance via the aminoglycoside 3′ acetyltransferase gene (*aadA*), which is used for selection of the transformants. The plasmid also contains a constitutively expressed mCherry (mCh) fluorescent reporter driven by the *groESL* promoter, which is used for microscopic evaluation of inclusion formation and overall bacterial growth. The resultant plasmids p2tk2_Spec_SW2mCh(gro)-CcdA-TetCcdB (Fig. 3A) and p2tk2_Spec_SW2mCh(gro)-MvpA-TetMvpT (Fig. 3B) were transformed into *C. trachomatis* serovar L2 (CTL2) using calcium-competent transformation, as previously described (34). The resulting transformants are referred to as CTL2 p2tk2_Spec_-CcdAB and CTL2 p2tk2_Spec_-MvpAT, respectively.

### The engineered CcdAB TA system does not efficiently inhibit *C. trachomatis* growth upon CcdB toxin induction

To test the ability of toxin CcdB to inhibit the proliferation of *C. trachomatis*, HeLa cells were infected with CTL2 p2tk2_Spec_-CcdAB. Infection with a strain expressing mCherry only served as a control. Infection and two subsequent passages were conducted in the absence (−aTc) or presence (+aTc) of aTc, and all passages were conducted in the presence of spectinomycin. Forty-eight hours after the initial infection (P0), after the first passage (P1), and after the second passage (P2), *C. trachomatis* inclusions were visualized and imaged using fluorescent microscopy (Fig. 3C). The number of inclusions was quantified for all strains, conditions, and time points. For each strain at a given passage, the number of inclusions in the +aTc condition was normalized relative to the number of inclusions in the −aTc condition. In the presence of aTc, a steady number of infectious bacteria were recovered from the control strain expressing mCherry only (Fig. 3D, gray bars). A similar result was observed with *C. trachomatis* encoding the CcdAB TA system, both with and without aTc (Fig. 3D, purple bars). Although there was a trend toward a decrease in recovered infectious progeny after each passage, this was not statistically significant, suggesting that in comparison to *E. coli*, expression of the CcdB toxin is not effective at killing *C. trachomatis*.

This result potentially indicates differences in susceptibility to the CcdB toxin between *E. coli* and *C. trachomatis*. The CcdB toxin inhibits bacterial proliferation through the inhibition of DNA gyrase via specific binding of CcdB to the DNA gyrase subunit A, GyrA (24, 35). Despite GyrA being highly conserved across bacterial species (36), an alignment of the amino acid sequences of *E. coli* GyrA and *C. trachomatis* GyrA revealed only 44.2% identity and 62.6% similarity between the two gyrase subunits (Fig. S2). Additionally, a significant portion of the GyrA subunit present in *E. coli* (amino acids 410–444 and 865–875) was absent in the *C. trachomatis* GyrA. The arginine (Arg) residue at position 462, which has been reported as essential for CcdB binding to GyrA (23), is not included in these deletions and conserved between *E. coli* and *C. trachomatis* (Fig. S2, Arg 428 in *C. trachomatis*). AlphaFold modeling of the GyrA proteins from *E. coli* and *C. trachomatis* individually revealed that while the overall tertiary structure of both proteins is conserved (Fig. S3A and B), the internal deletion of amino acids 410–444 upstream of the key Arg residue in *C. trachomatis* GyrA caused alterations in this region of the predicted *C*. trachomatis GyrA structure compared to *E. coli* GyrA (Fig. S3A and B). AlphaFold modeling of the GyrA-CcdB interaction revealed that, as previously reported, CcdB interacted with the region of *E. coli* GyrA containing Arg 462 (Fig. S3C); however, a similar interaction was not predicted between *C. trachomatis* GyrA and the region containing Arg 428 (Fig. S3D). These results further support the hypothesis that CcdB is inactive in *C. trachomatis* because it fails to interact with its GyrA target.

Another potential explanation for the difference in CcdB-mediated killing between *E. coli* and *C. trachomatis* could be the efficacy of toxin production. Although we validated that the plasmid harboring the CcdB toxin did not accumulate mutations preventing the inducible expression of a wild-type toxin, the lack of available validated antibodies prevented us from testing CcdB protein expression. We also note that the *ccdB* ORF was not codon optimized for *C. trachomatis* to account for differences in preferred synonymous codons between *C. trachomatis* and *S. flexneri*. Since codon optimization would have affected 56% of the *ccdB* codons, it is possible that protein production would have been improved. However, the codon optimization of the *mvpT* ORF, which would have affected 67% of the codons, was not necessary to induce MvpT-dependent killing (see below).

Altogether, although we cannot rule out a defect in CcdB production, we favor the hypothesis that the GyrA-CcdB interaction is altered in *C. trachomatis*.

### The engineered MvpAT TA system inhibits *C. trachomatis* growth upon MvpT toxin induction

To test the ability of toxin MvpT to inhibit the proliferation of *C. trachomatis*, HeLa cells were infected with CTL2 p2tk2_Spec_-MvpAT in the absence or presence of aTc, and infectious progeny production was quantified over time as described above for Fig. 3D. In stark contrast to the *C. trachomatis* strain carrying the CcdAB TA system (Fig. 3D, purple bars), the *C. trachomatis* strain encoding the MvpAT TA system displayed a significant reduction in recovered infectious progeny production in the presence of aTc (Fig. 3E, blue bars), resulting in 80% and 90% reduction in bacterial viability after one and two passages under toxin-inducing conditions, respectively. These results indicate that the MvpT toxin can effectively prevent *C. trachomatis* growth.

### Future application of the MvpAT counterselection system for use in *C. trachomatis*

Altogether, our results indicate that the MvpAT TA system, specifically toxin MvpT, is a viable counterselection marker to use in *C. trachomatis*.

With our current design, at steady state, bacterial viability is ensured by the TetR-dependent repression of toxin expression combined with the constitutive expression of the antitoxin to sequester any toxin produced due to leaky expression under repressive conditions (Fig. 1). While this was sufficient to counteract the effect of the MvpT toxin, tighter regulation could be achieved by combining transcriptional regulation via the Tet inducible system with the translational control via theophylline inducible riboswitch (37).

MvpT toxin-mediated killing was achieved in the presence of 2 ng/mL of aTc to alleviate TetR repression of the Tet promoter. Higher aTc concentrations could potentially be used to maximize toxin expression and the toxin/anti-toxin ratio. However, although aTc does not have any antimicrobial activity at low concentrations, prolonged exposure at concentrations higher than 2 ng/mL may have low levels of antimicrobial activity against *C. trachomatis*. For example, we have noticed a decrease in the recovery of infectious progeny from wild-type *C. trachomatis* bacteria over two passages in the presence of 20 ng/mL (not shown). It would therefore be important to titrate aTc carefully in case of lab-to-lab variation in aTc potency.

A counterselection system, such as the MvpAT TA system, is a powerful tool to expand the ever-growing *C. trachomatis* genetic toolbox. Combined with the FRAEM/FLAEM methodology (10, 38), it would offer a two-step selection and counterselection process to increase the rate of isolating transformants that have undergone both episodes of homologous recombination. This may help in the recovery of mutants with delayed developmental cycles by eliminating any wild-type bacteria that might otherwise overgrow the population. In addition to the generation of knockout mutants, the MvpAT counterselection system could also facilitate knocking-in alleles of interest (39, 40).

Another application would be to use the counterselection marker to eliminate bacteria that have failed to lose a plasmid of interest from a mixed population. For example, the cassette flanked by loxP sites that replaces a gene of interest in FRAEM mutants can be excised by transformation with a plasmid carrying the CRE recombinase (38). Loss of the CRE plasmid is achieved by conducting several passages in the absence of selection, prior to clonal isolation of the resulting FLAEM mutant via plaque purification or limiting dilution (38). Adding the MvpAT TA system to the CRE plasmid would enable counterselection of the bacteria that have retained the CRE plasmid and only amplify the FLAEM mutants that have lost the CRE plasmid, thus potentially reducing the number of passages required to eliminate CRE plasmid-bearing bacteria from the mixed population. A similar approach may be taken for transient expression of any protein of interest for which prolonged exposure may be detrimental to the bacteria, such as the C9 transposase used for transposition mutagenesis (7). One limitation with the current systems is that both MvpT toxin and C9 transposase expression are controlled by the Tet system, resulting in functional antagonism. However, it would be possible to use aTc to drive expression of the MvpT toxin and control translation of the C9 transposase via the theophylline inducible riboswitch (37) to allow for independent regulation of MvpT and C9 transposase production.

### Conclusions

*C. trachomatis* is responsible for the most commonly reported STI in the United States and one of the most common STIs globally. The identification of key bacterial factors that could serve as potential drug or vaccine targets has been significantly hindered by the obligate intracellular lifestyle of the bacteria and the technical challenges associated with genetic manipulation. Since a reliable and reproducible calcium-competent transformation method became available 14 years ago (13), we have witnessed the long-overdue expansion of the genetic tools for manipulating *C. trachomatis*. Despite this progress, the genetic toolkit for *C. trachomatis* remains limited compared to other bacterial systems, and many existing tools are still inefficient. By developing the first counterselection marker for use in *C. trachomatis*, we provide an additional genetic tool that enhances our ability to manipulate this pathogen. This advancement will not only accelerate functional studies of virulence factors but also directly support the discovery of novel therapeutic targets. Ultimately, this progress will translate to more effective strategies for preventing *Chlamydia* infections. Finally, the value of the system presented here extends to other obligate intracellular pathogens for which genetic tools are also limited.

## MATERIALS AND METHODS

### Cell lines and bacterial strains

HeLa cells (CCL-2) were obtained from American Type Culture Collection (ATCC) and cultured at 37°C with 5% CO_2_ in high-glucose Dulbecco’s modified Eagle’s medium (Invitrogen) supplemented with 10% heat-inactivated fetal bovine serum (Invitrogen). *C. trachomatis* lymphogranuloma venereum type II was obtained from the ATCC (L2/434/Bu VR-902B). *C. trachomatis* expressing mCherry (mCh WT) under the control of the *groESL* promoter was described previously (34). Plasmids used for *C. trachomatis* transformation were prepared from *E. coli* GM2163 (*dam*^−^ and *dcm*^−^). *C. trachomatis* propagation, infection, and transformation were performed as previously described (34, 41). All *C. trachomatis* strains were plaque purified.

### Plasmid construction

Restriction enzymes and T4 DNA ligase were obtained from New England BioLabs (Ipswich, MA). PCR was performed using Herculase DNA polymerase (Stratagene). PCR primers were obtained from Integrated DNA Technologies. Primers and cloning strategies are described in Table S1 and detailed below. All clonings were performed in *E. coli* DH5α.

### Construction of p2tk2_Amp_-CcdA-Tet-CcdB

A two-step cloning was conducted to construct p2tk2_Amp_-CcdA-Tet-CcdB. In Step 1, DNA fragments corresponding to the *incS* Promoter (PCR A) were amplified by PCR from *C. trachomatis* gDNA using primers *incS*prom KpnI 5 and *incS*promCcdASf 3. A DNA fragment corresponding to the CcdA toxin (PCR B) was amplified from *Shigella flexneri* 2457T gDNA using primers *incS*promCcdASf 5 and CcdASfinSterm 3. A DNA fragment corresponding to the *incS* terminator (PCR C) was amplified from *C. trachomatis* gDNA using primers CcdASfinSterm 5 and *incS*term Nco 3. A DNA fragment (PCR D) was amplified by overlapping PCR using PCR A, B, and C as templates and primers *incS*prom KpnI 5 and *incS*term Nco 3. Fragment D was cloned into pGEX2TK2_amp_ (42) at the KpnI/NcoI sites to generate p2tk2_Amp_-CcdA. In Step 2, DNA fragments corresponding to the *tet* repressor (*tetR*) and *tetA* promoter (*tetA^P^*) (PCR A) were amplified by PCR from the p2tk2_Spec_-TetR*tetA*PincV-3F-*incD* term (43) using primers tetR Nco 5 2 and TetCcdBSf 3. DNA fragments corresponding to the CcdB toxin (PCR B) were amplified by PCR from *Shigella flexneri* 2457T gDNA using primers TetCcdBSf 5 and CcdBSfinDterm 3. DNA fragments corresponding to the *incS* terminator (PCR C) were amplified using primers CcdBSf*incD*term 5 and *incD*term Not 3. A DNA fragment (PCR D) was amplified by overlapping PCR using PCR A, B, and C as templates and primers tetR Nco 5 2 and *incD*term Not 3. Fragment D was cloned into p2tk2_Amp_-CcdA at the NcoI/NotI sites to generate p2tk2_Amp_-CcdA-Tet-CcdB.

### Construction of p2tk2_Amp_-MvpA-Tet-MvpT

A two-step cloning was conducted to construct p2tk2_Amp_-MvpA-Tet-MvpT. In Step 1, DNA fragments corresponding to the *incS* promoter (PCR A) were amplified by PCR from *C. trachomatis* gDNA using primers *incS*prom KpnI 5 and *incS*promMvpASf 3. A DNA fragment corresponding to the MvpA toxin (PCR B) was amplified from *Shigella flexneri* 2457T gDNA using primers incSpromMvpASf 5 and MvpASfinSterm 3. A DNA fragment corresponding to the *incS* terminator (PCR C) was amplified from *C. trachomatis* gDNA using primers MvpASfinSterm 5 and *incS*term Nco 3. A DNA fragment (PCR D) was amplified by overlapping PCR using PCR A, B, and C as templates and primers *incS*prom KpnI 5 and *incS*term Nco 3. Fragment D was cloned into pGEX2TK2_amp_ (42) at the KpnI/NcoI sites to generate p2tk2_Amp_-MvpA. In Step 2, DNA fragments corresponding to the *tet* repressor (*tetR*) and *tetA* promoter (*tetA^P^*) (PCR A) were amplified by PCR from p2tk2_Spec_-TetR*tetA*PincV-3F-*incD*term (43) using primers tetR Nco 5 2 and TetMvpTSf 3. DNA fragments corresponding to the MvpT toxin (PCR B) were amplified by PCR from *Shigella flexneri* 2457T gDNA using primers TetMvpTSf 5 and MvpTSfinDterm 3. DNA fragments corresponding to the *incS* terminator (PCR C) were amplified using primers MvpTSf*inD*term 5 and *incD*term Not 3. A DNA fragment (PCR D) was amplified by overlapping PCR using PCR A, B, and C as templates and primers tetR Nco 5 2 and *incD*term Not 3. Fragment D was cloned into p2tk2_Amp_-MvpA at the NcoI/NotI sites to generate p2tk2_Amp_-MvpA-Tet-MvpT.

### Construction of p2tk2_Spec_SW2mCh(gro)-CcdA-Tet-CcdB

CcdA-Tet-CcdB was excised from p2tk2_Amp_-CcdA-Tet-CcdB using the KpnI/NotI sites and cloned into p2tk2_Spec_SW2mCh(gro) KpnI/NotI sites to generate p2tk2_Spec_SW2mCh(gro)-CcdA-Tet-CcdB.

### Construction of p2tk2_Spec_SW2mCh(gro)-MvpA-Tet-MvpT

MvpA-Tet-MvpT was excised from p2tk2_Amp_-MvpA-Tet-MvpT using the KpnI/NotI sites and cloned into p2tk2_Spec_SW2mCh(gro) KpnI/NotI sites to generate p2tk2_Spec_SW2mCh(gro)- MvpA-Tet-MvpT.

### Quantification of conditional toxin-mediated growth inhibition in *E. coli*

At 0 h, liquid cultures of DH5α p2tk2_Amp_-CcdA-Tet-CcdB and DH5α p2tk2_Amp_-MvpA-Tet-MvpT were diluted to an OD_600_ value of 0.05 in 50 mL of Luria broth media (Fisher #BP1426-2, composition tryptone 10 g/L, yeast extract 5 g/L, and sodium chloride 10 g/L). The liquid DH5α cultures were incubated on a rotator at 200 rpm at 37°C with ampicillin (final: 100 µg/mL) and the presence or absence of aTc (final: 200 ng/mL). The OD_600_ was measured again 8 h after infection.

### Quantification of conditional toxin-mediated growth inhibition in *C. trachomatis*

Two wells of a 24-well plate seeded with HeLa cells were infected with mCh WT, CTL2 p2tk2_Spec_SW2mCh(gro)-CcdA-Tet-CcdB (CcdAB), or CTL2 p2tk2_Spec_SW2mCh(gro)-MvpA-Tet-MvpT (MvpAT) at a multiplicity of infection of 0.5 in 2 ng/mL aTc (+aTc) or no aTc (−aTc) (=P0). Three images per well were taken at 48 h post-infection with the Nikon TE2000 microscope equipped with a Hamamatsu Orca ER Digital CCD Camera and a ×10 objective. Following imaging, cells were lysed in 100 μL sterile water, and fivefold dilutions of the lysates were used to infect fresh HeLa cell monolayers seeded in 96-well plates in the presence of spectinomycin (500 µg/mL) and the presence or absence of aTc, respectively (=P1). Forty-eight hours after the first passage, the same Nikon imaging and passaging protocol as P1 was repeated (=P2). Forty-eight hours after the second passage, the same Nikon imaging procedure was repeated, and the plates were aspirated. For each passage, the number of inclusions per image was quantified manually and averaged. For each *C. trachomatis* strain, the average inclusions in the +aTc condition were normalized to the average inclusions in their respective −aTc condition (avg. +aTc inclusions / avg. quantity −aTc inclusions) at each time point.

### Statistics

Each experiment was performed in triplicate. The SEM for the triplicate is shown. The graphs were generated using GraphPad Prism 10. The appropriate statistical tests were used and are indicated in the figure legends.

## SUPPLEMENTAL MATERIAL

Fig. S1 iai.00537-25-s0001.tif Sequence alignment and structure of tRNAfMet from *C. trachomatis*, *E. coli*, and *S. flexneri*.

Fig. S2 iai.00537-25-s0002.tif Amino acid alignment of the GyrA subunit of *E. coli* and *C. trachomatis* gyrase.

Fig. S3 iai.00537-25-s0003.tif Three-dimensional AlphaFold modeling of GyrA in complex with CcdB.

Legends iai.00537-25-s0004.docx Supplemental figure legends.

Table S1 iai.00537-25-s0005.docx Primers and templates used in plasmid design.

ASM does not own the copyrights to Supplemental Material that may be linked to, or accessed through, an article. The authors have granted ASM a non-exclusive, world-wide license to publish the Supplemental Material files. Please contact the corresponding author directly for reuse.
