# Supplementary figures and images for: Generation of a toxin/antitoxin-based counterselection marker for Chlamydia trachomatis
Source: Infect Immun. 2025 Nov 18;93(12):e00537-25. doi: 10.1128/iai.00537-25 (PMC12707109; doi:10.1128/iai.00537-25)

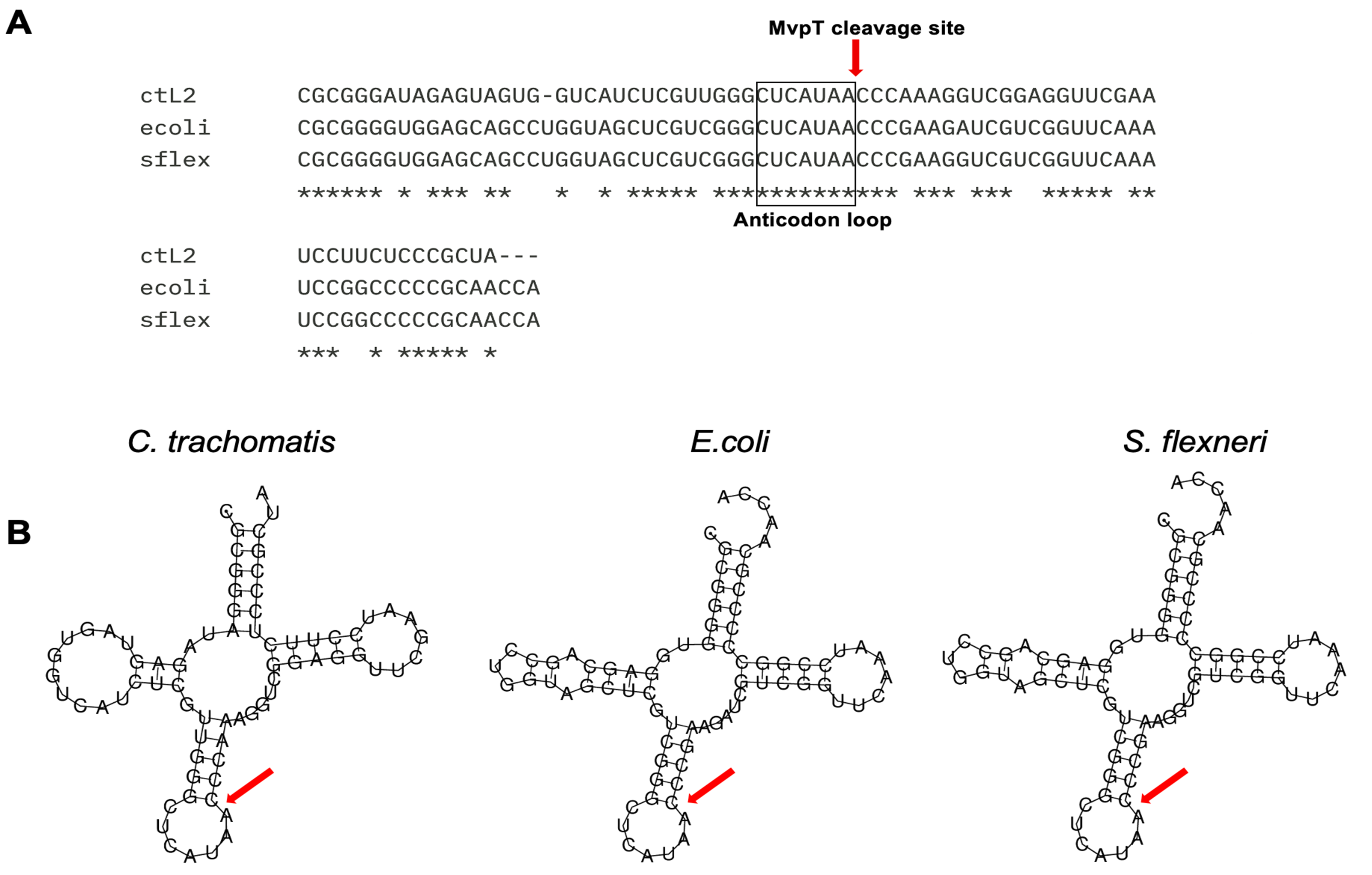

Supplement: Fig. S1 — Sequence alignment and structure of tRNAfMet from C. trachomatis, E. coli, and S. flexneri. [file iai.00537-25-s0001.tif]

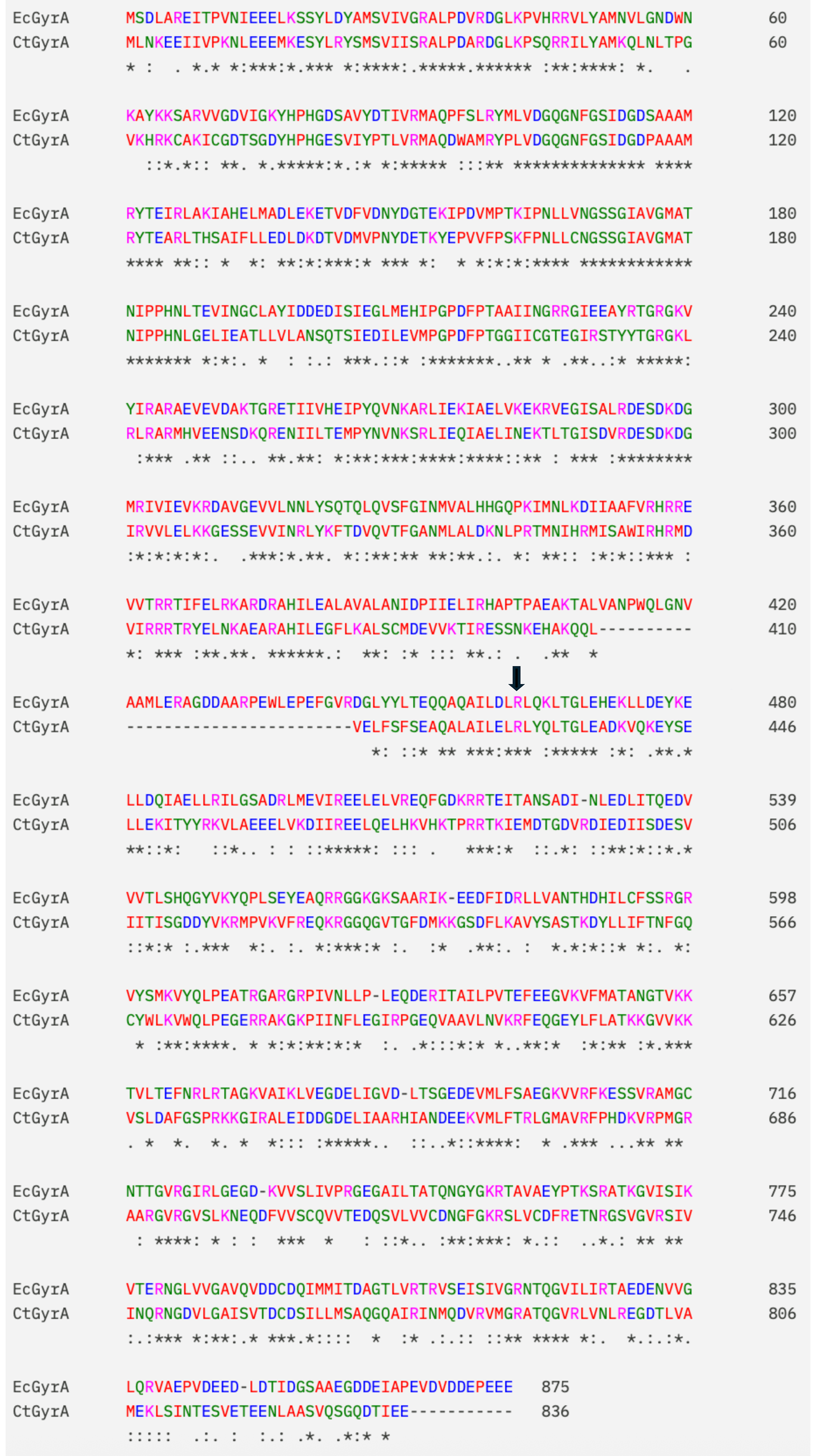

Supplement: Fig. S2 — Amino acid alignment of the GyrA subunit of E. coli and C. trachomatis gyrase. [file iai.00537-25-s0002.tif]

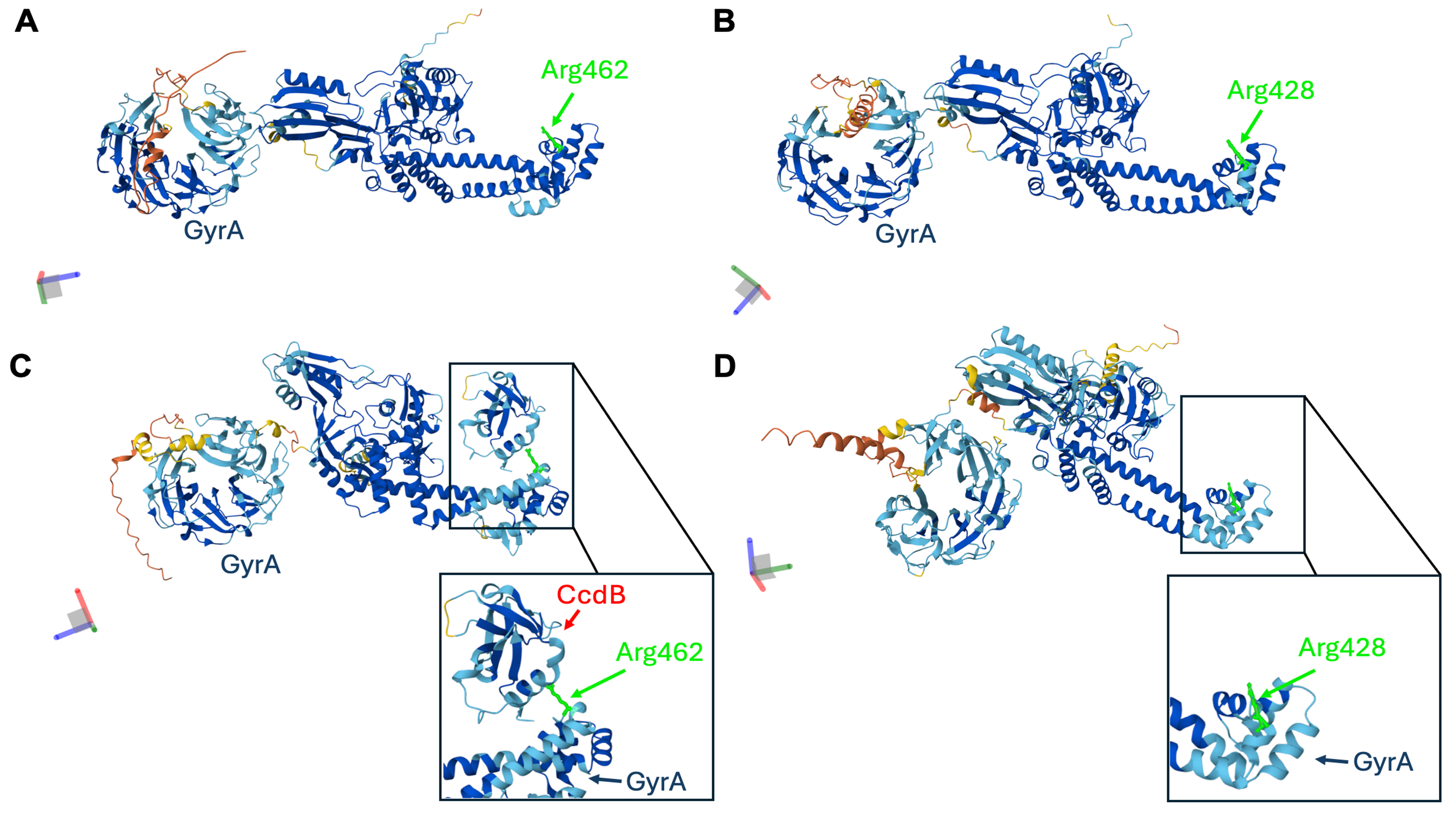

Supplement: Fig. S3 — Three-dimensional AlphaFold modeling of GyrA in complex with CcdB. [file iai.00537-25-s0003.tif]
