# Supplementary material for: Generation of a toxin/antitoxin-based counterselection marker for Chlamydia trachomatis
Source: Infect Immun. 2025 Nov 18;93(12):e00537-25. doi: 10.1128/iai.00537-25 (PMC12707109; doi:10.1128/iai.00537-25)
Supplement: Legends — Supplemental figure legends. [file iai.00537-25-s0004.docx]

**Supplementary Figures Legends:**

**Supplemental Figure 1. Sequence alignment and structure of tRNA^fMet^ from *C. trachomatis*, *E. coli*, and *S. flexneri*.** (**A**) Sequence alignment of the tRNA^fMet^ from *C. trachomatis* L2 (ctL2), *E. coli* K12(ecoli), and *S. flexneri* (sflex). The conserved sequence for the anticodon loop is boxed, and the conserved MvpT cleavage site is indicated by a red arrow. (**B**) RNAfold minimum free energy predictions of the tRNA^fMet^ secondary structure from *C. trachomatis* L2, *E. coli* K12, and *S. flexneri*. The MvpT cleavage site is indicated by a red arrow.

**Supplemental Figure 2. Amino acid alignment of the GyrA Subunit of *E. coli* and *C. trachomatis* gyrase.** Alignment of GyrA Subunit of *E. coli* gyrase (EcGyrA) and *C. trachomatis* (CtGyrA) using Clustal Omega. The conserved arginine residue (R) is indicated with black arrow.

**Supplemental Figure 3.** **Three-dimensional AlphaFold modelling of GyrA in complex with CcdB. (A-B)** AlphaFold prediction of the structure of the *E. coli* (A) or *C. trachomatis* (B) GyrA subunit. The conserved arginine residue important for the GyrA-ccdB interaction is shown in green. **(C-D)** AlphaFold prediction of the structure of the *E. coli* (C) or *C. trachomatis* (D) GyrA subunit in complex with CcdB, with zoomed in view of the interaction interface between CcdB and GyrA via arginine residue 462 in *E. coli* (C) and of the lack of interaction with arginine residue 428 in *C. trachomatis* (D).
