# Supplementary material for: Generation of a toxin/antitoxin-based counterselection marker for Chlamydia trachomatis
Source: Infect Immun. 2025 Nov 18;93(12):e00537-25. doi: 10.1128/iai.00537-25 (PMC12707109; doi:10.1128/iai.00537-25)
Supplement: Table S1 — Primers and templates used in plasmid design. [file iai.00537-25-s0005.docx]

**Supplemental Table 1: Primers and templates used in plasmid design**

| **Cloning:** | **PCR:** | **Primer Names:** | **Primer Sequences:** | **Template:** |
| --- | --- | --- | --- | --- |
| p2tk2_Am_p-CcdA-  TetCcdB | **Step 1:** | | | |
|  | PCR A | *incS*prom KpnI 5 | GGTGGTACCaaacgagtttttttctaagag | *C. trachomatis*  gDNA |
|  |  | incSpromCcdASf 3 | GACAGTAATACGCTGCTTCATactttcctgaaaaactatttttg |  |
|  | PCR B | *incS*promCcdASf 5 | caaaaatagtttttcaggaaagtATGAAGCAGCGTATTACTGTC | *Shigella flexneri*  2457T gDNA |
|  |  | CcdASfinSterm 3 | gctttagcagccctcctttttTCACCAGTCCCTGTTCTCATC |  |
|  | PCR C | CcdASfinSterm 5 | GATGAGAACAGGGACTGGTGAaaaaaggagggctgctaaagc | *C. trachomatis*  gDNA |
|  |  | *incS*term Nco 3 | CCACCATGGacaagtctgttctatacatc |  |
|  | PCR D | *incS*prom KpnI 5 | GGTGGTACCaaacgagtttttttctaagag | Fragments A, B, C |
|  |  | *incS*term Nco 3 | CCACCATGGacaagtctgttctatacatc |  |
|  | **Step 2:** | | | |
|  | PCR A | tetR Nco 5 2 | CCACCATGGTTAAGACCCACTTTCAC | p2TK2_Spec_TetR*tetA*PincV-3FincDterm |
|  |  | TetCcdBSf 3 | GGCGTAAACCTTAAACTGCATttcacttttctctatcactg |  |
|  | PCR B | TetCcdBSf 5 | cagtgatagagaaaagtgaaATGCAGTTTAAGGTTTACGCC | *Shigella flexneri*  2457T gDNA |
|  |  | CcdBSfinDterm 3 | cgcgaatcacatgtcatccTTATATTCCCCAGAACATCAGG |  |
|  | PCR C | CcdBSfinDterm 5 | CCTGATGTTCTGGGGAATATAAggatgacatgtgattcgcg | *C. trachomatis*  gDNA |
|  |  | *incD*term Not 3 | GCGGCGGCCGCgtcttaggagctttttgc |  |
|  | PCR D | tetR Nco 5 2 | CCACCATGGTTAAGACCCACTTTCAC | Fragments A, B, C |
|  |  | *incD*term Not 3 | GCGGCGGCCGCgtcttaggagctttttgc |  |
| p2tk2_Am_p -  MvpA-  TetMvpT | **Step 1:** | | | |
|  | PCR A | *incS*prom KpnI 5 | GGTGGTACCaaacgagtttttttctaagag | *C. trachomatis*  gDNA |
|  |  | *incS*promMvpASf 3 | GAAATACGGTGGTTTCCATactttcctgaaaaactatttttg |  |
|  | PCR B | *incS*promMvpASf 5 | caaaaatagtttttcaggaaagtATGGAAACCACCGTATTTC | *Shigella flexneri*  2457T gDNA |
|  |  | MvpASfinSterm 3 | gctttagcagccctcctttttTCAGAATGACTCCCTTTCTTGC |  |
|  | PCR C | MvpASfinSterm 5 | GCAAGAAAGGGAGTCATTCTGAaaaaaggagggctgctaaagc | *C. trachomatis*  gDNA |
|  |  | *incS*term Nco 3 | CCACCATGGacaagtctgttctatacatc |  |
|  | PCR D | *incS*prom KpnI 5 | GGTGGTACCaaacgagtttttttctaagag | Fragments A, B, C |
|  |  | *incS*term Nco 3 | CCACCATGGacaagtctgttctatacatc |  |
|  | **Step 2:** | | | |
|  | PCR A | tetR Nco 5 2 | CCACCATGGTTAAGACCCACTTTCAC | p2TK2_Spec_TetR*tetA*Pinc V-3F*incD*term |
|  |  | TetMvpTSf 3 | CGAGCATAAACTTCAGCATttcacttttct ctatcactg |  |
|  | PCR B | TetMvpTSf 5 | cagtgatagagaaaagtgaaATGCTGAAGTTT ATGCTCG | *Shigella flexneri*  2457T gDNA |
|  |  | MvpTSfinDterm 3 | cgcgaatcacatgtcatccTCAGCTCCAGTCT TCAGTTCTCAG |  |
|  | PCR C | MvpTSfinDterm 5 | CTGAGAACTGAAGACTGGAGCTGAggatgacatgtgattcgcg | *C. trachomatis*  gDNA |
|  |  | *incS*term Nco 3 | CCACCATGGacaagtctgttctatacatc |  |
|  | PCR D | tetR Nco 5 2 | CCACCATGGTTAAGACCCACTTTCAC | Fragments A, B, C |
|  |  | *incD*term Not 3 | GCGGCGGCCGCgtcttaggagctttttgc |  |
